# An Interval of the Obesity QTL *Nob3.38* within a QTL Hotspot on Chromosome 1 Modulates Behavioral Phenotypes

**DOI:** 10.1371/journal.pone.0053025

**Published:** 2013-01-04

**Authors:** Heike Vogel, Dirk Montag, Timo Kanzleiter, Wenke Jonas, Daniela Matzke, Stephan Scherneck, Alexandra Chadt, Jonas Töle, Reinhart Kluge, Hans-Georg Joost, Annette Schürmann

**Affiliations:** 1 Departments of Pharmacology, Experimental Diabetology, German Institute of Human Nutrition Potsdam-Rehbruecke, Nuthetal, Germany; 2 Research Group Neurogenetics, Leibniz Institute for Neurobiology, Magdeburg, Germany; 3 German Diabetes Center, Leibniz Center for Diabetes Research at Heinrich-Heine-University Düsseldorf, Düsseldorf, Germany; 4 Department of Molecular Genetics, German Institute of Human Nutrition Potsdam-Rehbruecke, Nuthetal, Germany; University of Chicago, United States of America

## Abstract

A region on mouse distal chromosome 1 (Chr. 1) that is highly enriched in quantitative trait loci (QTLs) controlling neural and behavioral phenotypes overlaps with the peak region of a major obesity QTL (*Nob3.38*), which we identified in an intercross of New Zealand Obese (NZO) mice with C57BL/6J (B6). By positional cloning we recently identified a microdeletion within this locus causing the disruption of *Ifi202b* that protects from adiposity by suppressing expression of *11β-Hsd1*. Here we show that the *Nob3.38* segment also corresponds with the QTL rich region (*Qrr1*) on Chr. 1 and associates with increased voluntary running wheel activity, Rota-rod performance, decreased grip strength, and anxiety-related traits. The characterization of a subcongenic line carrying 14.2 Mbp of *Nob3.38* with a polymorphic region of 4.4 Mbp indicates that the microdeletion and/or other polymorphisms in its proximity alter body weight, voluntary activity, and exploration. Since 27 out of 32 QTL were identified in crosses with B6, we hypothesized that the microdeletion and or adjacent SNPs are unique for B6 mice and responsible for some of the complex *Qrr1*-mediated effects. Indeed, a phylogenic study of 28 mouse strains revealed a NZO-like genotype for 22 and a B6-like genotype for NZW/LacJ and 4 other C57BL strains. Thus, we suggest that a *Nob3.38* interval (173.0–177.4 Mbp) does not only modify adiposity but also neurobehavioral traits by a haplotype segregating with C57BL strains.

## Introduction

Obesity is the major risk factor for cardiovascular disease, insulin resistance, and type 2 diabetes [1], [2]. Epidemiological studies indicate that obesity is significantly associated with a higher incidence of neuropsychiatric symptoms [3]–[6], depressive symptoms [7]–[9], history of depression [10], and measures of psychological distress [11], [12]. Simon et al. [13] demonstrated that obesity is associated with an approximately 25% increase in odds of mood and anxiety disorders in a nationally representative sample of the US household population. However, these epidemiological studies only allow highlighting associations between symptoms but they cannot clarify a mechanistic link between increased body weight and mental state. For instance, a majority of studies evaluating associations of depression and obesity find a prospective relationship between eating disturbances and depression [14], but the relationship is not unidirectional. Thus depression may be both cause and consequence of obesity [15] and interactions between both symptoms are more complicated because they involve psychosocial factors.

Obesity is an inherited disorder, and candidate gene approaches as well as genome-wide association studies have identified several loci that associate with increased body weight [16]. By generating and characterizing backcross and F2 generations of the obese NZO (New Zealand Obese) and lean mice such as SJL (Swiss Jim Lambert) or C57BL/6 (B6), we identified several quantitative trait loci (QTL) for the metabolic syndrome and type 2 diabetes [17]–[21]. Subsequently, critical fragments were defined by interval-specific introgressions to the B6 background resulting in the positional cloning of the obesity gene *Tbc1d1* [22] and the diabetes susceptibility gene *Zfp69* [23]. With this approach, we recently discovered a microdeletion on chromosome 1 of B6 mice causing the disruption of *Ifi202b* and an altered expression of several other genes and suggested that these alterations protect B6 mice from adiposity [24]. Interestingly, the peak of *Nob3.38* including the microdeletion overlaps with a QTL hotspot (*Qrr1*) on Chr. 1 that is associated not only with metabolic but also with behavioral traits [25]. These traits include open field activity, fear conditioning, rearing behavior and several other measures of emotionality. Since 27 out of 32 QTL on distal Chr. 1 were found in crosses with B6 as one breeding partner, we hypothesize that a B6 specific alteration is responsible for the complex phenotype. This could be a recently identified microdeletion and/or polymorphisms in its proximity. Here, we demonstrate that *Nob3.38* associates with differences in voluntary running wheel activity, Rota-rod performance, and an altered swimming behavior. We furthermore show that the microdeletion is specific for most C57 strains whereas other mice (e.g. C3H/HeJ or FVB/NJ) carry the NZO genotype in this region.

## Methods

### Animals

The animals were kept in accordance with the NIH guidelines for the care and use of laboratory animals, and all experiments were approved by the ethics committee of the State Agency of Environment, Health and Consumer Protection (State of Brandenburg, Germany). Female NZO mice from our own colony (NZO/HIBomDife), NZB/OlaHsd (Harlan Winkelmann, Paderborn, Germany), C57BL/6J, FVB/NCrl (Charles River, Sulzfeld, Germany), 129S6/SvEvTac, SJL/NBom (Taconic, M+B, Ry, Denmark), C3H/HeJ, DBA/2J, BALB/cJ, CAST/EiJ, C57BL/10J (The Jackson Laboratory, Bar Harbor, Maine, USA), and congenic lines [21] were used throughout. Mice were housed at a temperature of 22°C with a 12∶12 hours light-dark cycle (lights on at 6:00 a.m.) in type II or type III macrolon cages with soft wood bedding. Standard chow (ssniff, Soest, Germany; Art. No. V153xR/M-H) contained 19% (w/w) protein, 3.3% fat, and 54.1% carbohydrates, with 26, 10, and 74% of total digestible energy (12.8 kJ/g) from protein, fat, and carbohydrates, respectively.

### Breeding strategy and genotyping

Breeding of the congenic line B6.NZO-*Nob3.38* was previously described [21]. Additional recombinant congenic strains (RCS) were generated by backcrossing of male B6.NZO-*Nob3.38* with 6–12 B6 females [24]. For genotyping, DNA was prepared from mouse tails with a DNA isolation kit based on a salt precipitation method (InViTek, Berlin, Germany) and used for tests with polymorphic microsatellite markers. Microsatellites were genotyped by PCR with oligonucleotide primers obtained from MWG (Ebersberg, Germany), and microsatellite length was determined by non-denaturing polyacrylamide gel electrophoresis. For further fine mapping of the critical region single nucleotide polymorphisms (SNPs) were used and analysed by sequencing. Phenotypical characterization of congenic line B6.NZO-*Nob3.38* was performed in F6N6 generation and of the RCS-IX in F8N6. As previously described for the determination of the residual NZO genome founders of the N5 generation were genotyped for 114 polymorphic markers and they carried <10% NZO genome, resulting in <5% NZO genome in congenic line B6.NZO-*Nob3.38* and <1.5% in RCS-IX. None of the other significant or suggestive QTL identified in the original F2 cross were detected [21]. The congenic line B6.NZO-*Nob3.38* is defined by the microsatellite markers D1Mit202 and D1Mit209; congenic line RCS-IX by D1Mit143 and D1Mit115 (Table S1).

### Locomotor and running wheel activity

Locomotor activity was monitored after a 2 day adaptation period with an infrared detector (TSE InfraMot-Activity System, TSE, Bad Homburg, Germany). Mice were single housed and had free access to food and water. Voluntary running wheel activity was recorded with an automated running wheel system (TSE). Mice were habituated to the wheels and type III Macrolon cages for 2 days before the data collection period. The animals had free access to the running wheels as well as to food and water. Data were expressed as total number of revolutions per hour.

### Behavioral screening

For the behavioral analysis, sex- and age-matched mice were used (12 females and 11 males for each B6.NZO-*Nob3.38^N/N^* and B6.NZO-*Nob3.38^B/B^* line). For behavioral tests of the subcongenic line RCS-IX we used 5 females of *B/B* and 7 females of *N/N* genotype. During the light phase, mice were subjected to a series of behavioral tests [26], [27] by an experimenter not aware of the genotype. First, general parameters indicative of the health and neurological state were addressed following the neurobehavioral examination described by Whishaw and colleagues [28] and the tests of the primary screen of the SHIRPA protocol except startle response [29].

### Grip strength

Strength was measured with a high-precision force sensor to evaluate neuromuscular functioning (TSE).

### Light-dark avoidance

Anxiety-related behavior was tested by placing mice in a brightly lit compartment (250 lux, 25×25 cm) adjacent to a dark compartment (12.5×25 cm). The number of transitions between the compartments and the time spent within each were analyzed during 10 min. As a test for long term memory [30], animals were placed at the last day of testing again in the light-dark avoidance box. The latency to enter the dark compartment was measured and compared to the latency at the first time in the box.

### Rota-rod performance

Animals received two training sessions (3 h interval) on a Rota-rod apparatus (TSE) with increasing speed from 4 to 40 rpm for 10 min. After 4 days, mice were tested at 16, 24, and 32 rpm constant speed. The latency to fall off the rod was measured.

### Open field

Exploration was assessed by placing mice in the middle of a 50×50 cm area (for 15 min). Using the VideoMot 2 system (TSE), tracks were analyzed for path length, visits, and relative time spent in the central area (infield), in the area close to the walls (<10 cm, outfield), and in the corners, walking speed, latency to move, time moving or resting, number of stops and rests.

### O-Maze

Anxiety and exploration was also tested on an O-Maze (San Diego Instruments, CA, USA). Mice were placed in the center of an open area and their behavior during 5 min was recorded on videotape. The number of entries into the closed or open areas was counted. The time spent in these compartments and the distances traveled were determined using the VideoMot 2 system (TSE).

### Morris Water Maze

Spatial learning was assessed in the hidden platform version of the Morris task. Mice were allowed to swim until they found the platform or until 120 s had elapsed. The animals received 6 trails per day during 5 consecutive days with the platform positioned in the south-east quadrant during the first 3 days (total of 18 trials, acquisition phase), and in the opposite quadrant for the last 2 days (total of 12 trials, reversal phase). Trials 19 and 20 were defined as probe trials to analyze the precision of spatial learning. Trials were analyzed using the VideoMot 2 system (TSE) and Wintrack software [31].

### Expression analysis in mouse tissues by quantitative real-time PCR

Total RNA from white adipose tissue was extracted with the RNeasy Mini Kit (Qiagen, Hilden, Germany) according to the guideline of the manufacturer. Extraction of total RNA from skeletal muscle, brain, cortex, bulbus olfactorius, cerebellum, and hippocampus was performed with TRizol™ reagent (Invitrogen, Carlsbad, CA). First strand cDNA synthesis was prepared with 2.0 µg total RNA, random hexamer primer, and SuperscriptIII reverse transcriptase (Invitrogen). Quantitative real-time PCR was performed with an Applied Biosystems 7300 Real-time PCR system, with TaqMan Gene Expression Master Mix or SybrGreen Master Mix (Applied Biosystems, Darmstadt, Germany), 25 ng cDNA, and TaqMan Gene Expression Assays (Applied Biosystems) or oligonucleotides (MWG) (Table S1). Data were normalized referring to Livak and Schmittgen [32], whereas a β-actin expression assay was used as endogenous control.

### Sequencing

Sequencing of DNA was performed with a 3130×l Genetic Analyzer (Applied Biosystems) in combination with the BigDye Terminator v3.1 Cycle Sequencing Kit (Applied Biosystems). Sequence analysis was done by SeqScape software 2.5 (Applied Biosystems).

### Isolation of mitochrondria of skeletal muscle and determination of the mitochondrial capacity

Isolation of mitochondria from skeletal muscle of B6.NZO-*Nob3.38^B/B^* and B6.NZO-*Nob3.38^N/N^* mice was based on the procedure described by Wibom et al. [33]. Briefly, the muscle sample (*Tibialis anterior*, approx. 100–250 mg) was transferred in a tube containing ice cold isolation buffer (sucrose 100 mM, KCl 100 mM, Tris-HCl 50 mM, KH_2_PO_4_ 1 mM, EGTA 0.1 mM, BSA 0.2%, and pH 7.4) and cut into small pieces with scissors. After 2 min resting time the supernatant was removed and the pellet was cleaned with 1 ml isolation buffer. The step was repeated 3 times. After final removal of the supernatant, 1 ml of protease solution (11 U/mg) was added and the sample was homogenized for 2 min. The solution was centrifuged at 750×g for 10 min (4°C). The pellet was discarded and the supernatant centrifuged for 10 min at 10,000×g (4°C). The new pellet, containing the mitochondria, was washed in isolation buffer, recentrifuged (7,000×g for 4 min, 4°C), and carefully resuspended in suspension buffer, containing 225 mM mannitol, 75 mM sucrose, 10 mM Tris, 0.1 mM EDTA, pH 7.4 (initial weight of muscle tissue times 0.4 µl). The isolated mitochondria were packed in tinfoil and kept on ice until the respiration measurement by Seahorse XF24 (Seahorse Biosciences, North Billerica, MA, USA). Mitochondria were added to wells containing respiration buffer (220 mM mannitol, 70 mM sucrose, 5 mM MgCl_2_, 5 mM KH_2_PO_4_, 0.2% FA free BSA, 2 mM HEPES, 1 mM EGTA, 5.5 mM succinate, 2.2 µM rotenone, pH 7.2) to yield a final concentration of 2 µg of mitochondrial protein/500 µl (final volume). After measurement of basal respiration 1 mM ADP was added and designated as state 3 respiration, followed by the addition of oligomycin (2 µM, an ATP synthase inhibitor) to induce state 4+ respiration, a measure of mitochondrial uncoupling activity. State 5 respiration was induced with the mitochondrial uncoupler carbonyl cyanide 4-(trifluoromethoxy) phenylhydrazone (FCCP; 4 µM) to get maximum uncoupled electron transport. Finally an inhibitor of complex 3 (antimycin A; 4 µM) was added to block respiratory chain dependent respiration. The individual respiration state was calculated from the oxygen consumption slopes vs. time, and transformed to OCR (oxygen consumption rate) in units of pmol/min. The respiratory control ratio (RCR) was calculated by dividing the slope of the response of isolated mitochondria to state 3 respiration (presence of ADP) by slope of the response to oligomycin-induced state 4 respiration.

### Citrate synthase activity

Frozen muscle samples were homogenized in Tris-EDTA buffer and cleared supernatant after centrifugation was used for spectrophotometric assay. The conversion rate of acetyl-CoA and oxaloacetate to citrate and CoA-SH by citrate synthase is proportional to the coupled reaction of CoA-SH and DTNB to TNB, which was measured at 412 nm.

### Glucose uptake and fatty acid oxidation

Measurements of glucose transport and fatty acid oxidation were performed in isolated EDL (*Extensor digitorum longus*) and soleus muscles of B6.NZO-*Nob3.38^B/B^* and B6.NZO-*Nob3.38^N/N^* mice as previously described [22].

### Statistical analysis

Values are reported as means ± SE, unless otherwise noted. Differences between *B/B* and *N/N* genotypes were tested by two-tailed Student's t test. The behavioral data were analyzed by repeated measures, 1-way, and 2-way ANOVA (factors genotype and sex) and post hoc analysis with Scheffe's test (Statview Program, SAS Institute Inc., Cary, NC). The number of males and females for each genotype was identical permitting to analyze the pooled data when the interaction of genotype and sex was not significant. Expression levels determined by quantitative real-time PCR were compared by the nonparametric Kruskal-Wallis H test. A p-value smaller than 0.05 (p<0.05) was considered significant.

## Results

### The obesity locus Nob3.38 maps to a QTL hotspot associated with diverse metabolic and behavioral traits

**Figure 1:**
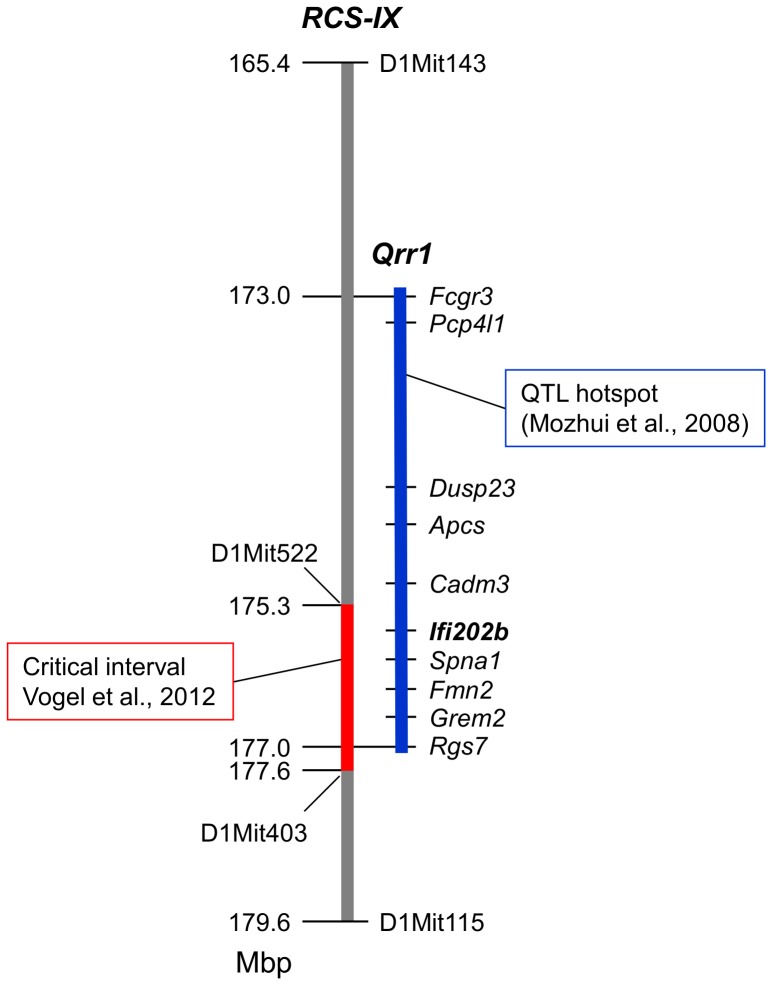
The peak region of the obesity QTL *Nob3* corresponds with the QTL hotspot *Qrr1*. The map depicts the critical interval recently identified to be responsible for differences in body weight of NZO and B6 allele carriers [24]. Genes located in the region on Chr. 1 and a selection of microsatellite markers used for genotyping of the subcongenic line RCS-IX are indicated. QTL rich region on Chr. 1 (*Qrr1*) extending from the *Fcgr3* to *Rgs7* gene is shown in blue (NCBI Build 37/mm9).

**Figure 2:**
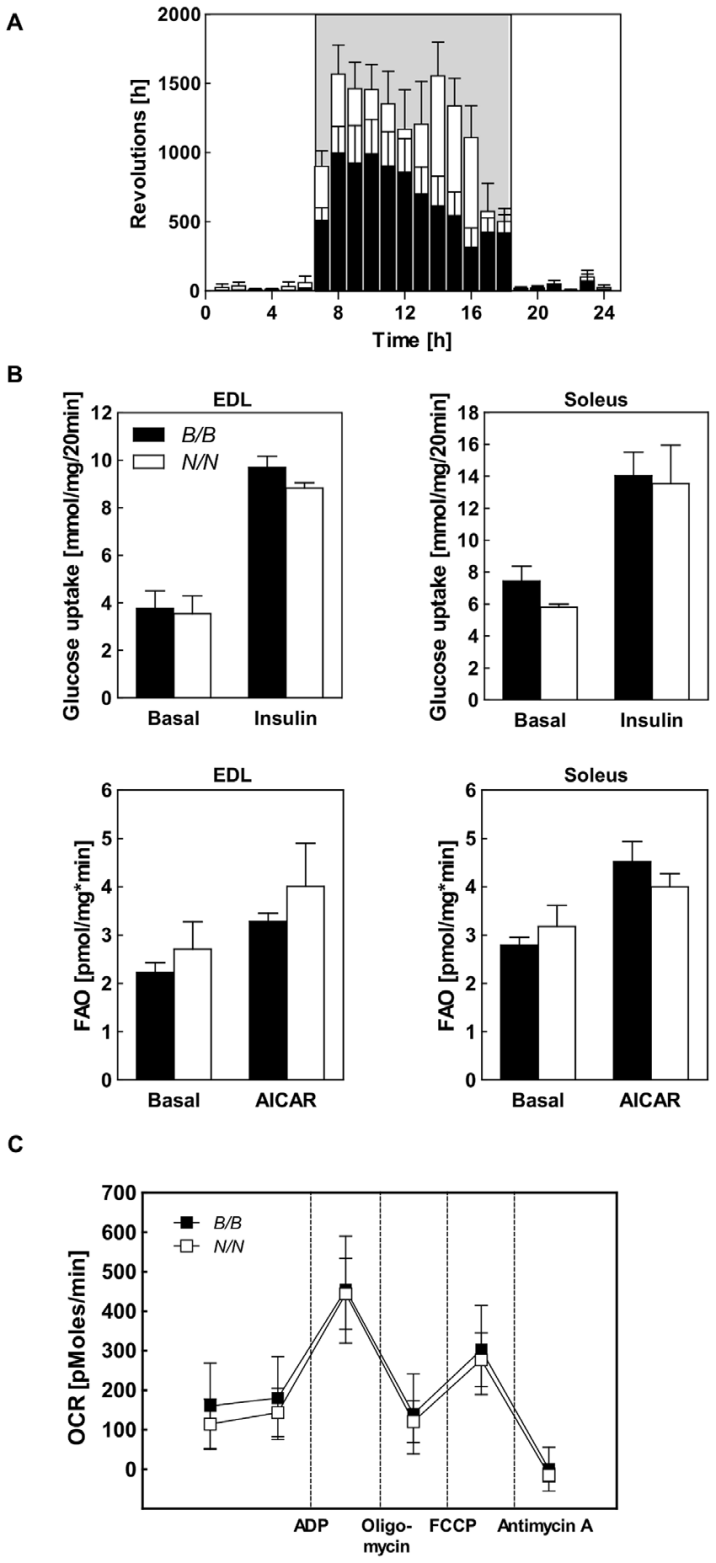
Characterization of voluntary activity and the metabolism of skeletal muscle of *Nob3.38^B/B^* and *Nob3.38^N/N^* mice. (A) Voluntary activity of *B/B* and *N/N* mice detected with a running wheel over 24 h (n = 13). (B) Glucose uptake into isolated skeletal muscle under basal and insulin-stimulated conditions (upper panel) and palmitate oxidation (lower panel) in isolated skeletal muscle under basal and AICAR stimulated conditions (EDL, left panels; Soleus, right panels) of *B/B* and *N/N* mice. (C) Characterization of mitochondrial respiration by determination of oxygen consumption rate of isolated mitochondria from skeletal muscles of *B/B* and *N/N* mice (n = 4–6).

The polymorphic region of the *Nob3.38* interval previously described to significantly increase body weight [21] maps to a region highly enriched with QTL, designated QTL-rich region on Chr 1 (*Qrr1*; Figure 1) [25]. The critical interval of *Nob3.38* comprising the adipogenic allele was defined by the markers D1Mit522 and D1Mit403 between 175.3 and 177.6 Mbp and the borders flanked by *Cadm3* and *Rgs7* (Figure 1) which maps within *Qrr1* (172–178 Mbp). *Qrr1* modifies complex traits such as body weight, blood glucose as well as several neuronal and behavioral phenotypes (Table S2) [25]. For 84% of the 32 QTL that were in linkage disequilibrium on distal Chr. 1, the B6 strain was used as breeding partner, suggesting that a variation in the B6 genome is responsible for the complex effects of *Qrr1*. Thus, we tested whether *Nob3.38*, in addition to modifying adiposity, body temperature, and energy expenditure [24], conferred some of the behavioral traits described for *Qrr1* [25], [33], [35]. Congenic mice carrying the adipogenic NZO allele of *Nob3.38* (B6.NZO-*Nob3.38^N/N^*) were studied in comparison to mice carrying the corresponding B6 allele (B6.NZO-*Nob3.38^B/B^*). In the home cage, the B6.NZO-*Nob3.38^B/B^* and B6.NZO-*Nob3.38^N/N^* mice did not show differences in locomotor activity [24]. However, when mice had access to a running wheel, the voluntary activity was elevated in carriers of the NZO alleles (Figure 2A). This increased activity was not a consequence of an altered muscle metabolism, since basal and insulin stimulated glucose transport, fatty acid oxidation in two types of muscle (Figure 2B), and mitochondrial respiration of the skeletal muscle (Figure 2C) were identical in B6.NZO-*Nob3.38^B/B^* and B6.NZO-*Nob3.38^N/N^* mice. Furthermore, citrate synthase activity (B6.NZO-*Nob3.38^B/B^*: 1.49±0.06 vs. B6.NZO-*Nob3.38^N/N^*: 1.44±0.092 mmol/min/µg protein) and the ratio between type-I and type-II fibres (data not shown) did not differ in muscles of B6 and NZO allele carriers, supporting our assumption that increased activity of B6.NZO-*Nob3.38^N/N^* mice is not the result of changes in muscle physiology.

**Figure 3:**
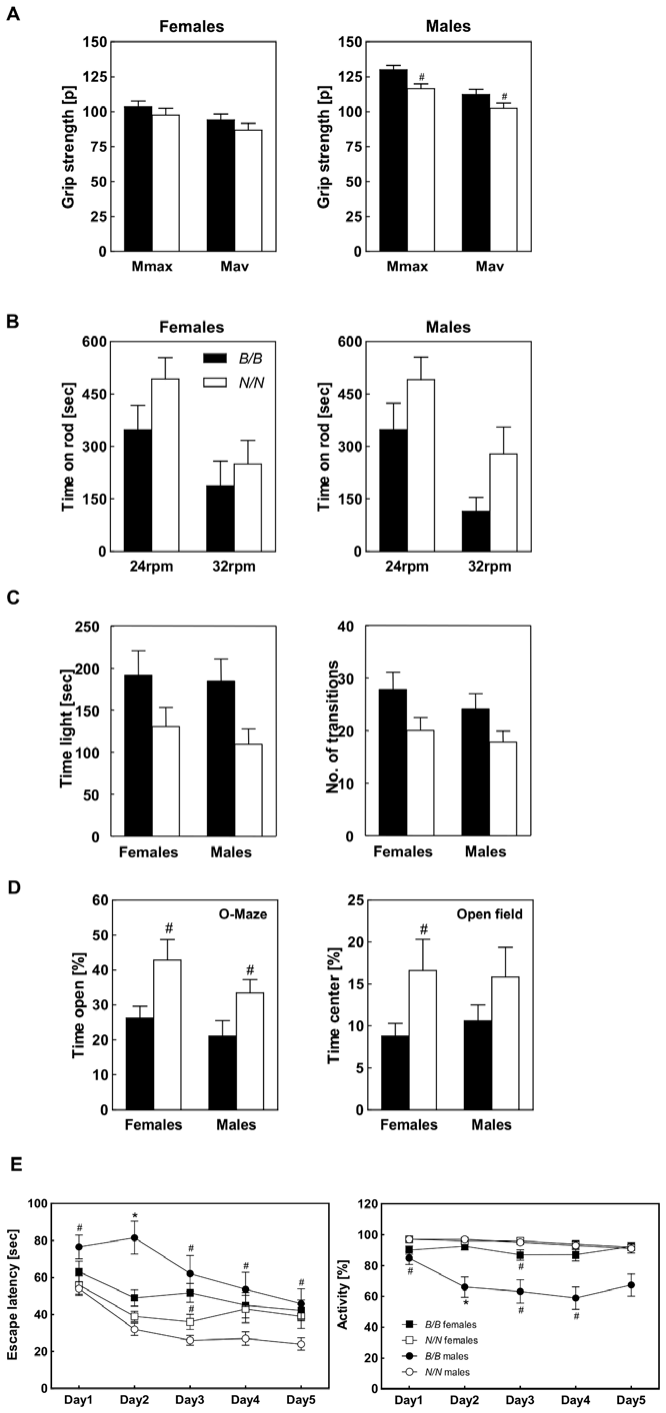
Characterization of the behavior of B6.NZO-*Nob3.38^B/B^* and B6.NZO-*Nob3.38^N/N^*. (A) Grip strength (maximum and average of 5 measurements), (B) Rota-rod performance at 24 and 32 rpm constant speed for 10 min, (C) time spent in illuminated compartments and number of transitions during light-dark avoidance test, (D) avoidance of the open areas of the O-Maze (left panel) and the center of the open field during the initial 5 min (right panel). (E) Spatial learning was assessed by the Morris Water Maze test as described in Material and Methods (n = 12). For the behavioral analysis, sex- and age-matched (10–12 weeks) mice were used (12 females and 11 males for each B6.NZO-*Nob3.38^N/N^* and B6.NZO-*Nob3.38^B/B^* line). (#p<0.05; *p<0.001).

In order to test whether *Nob3.38* affects neuronal functions which may also finally result in elevated running wheel activity, we assessed motor coordination and grip strength, exploration, anxiety, and learning related behaviors by Rota-rod, open field, O-Maze, light-dark avoidance, water maze, and fear conditioning paradigms with B6.NZO-*Nob3.38^B/B^* and B6.NZO-*Nob3.38^N/N^* mice. Although heavier, B6.NZO-*Nob3.38^N/N^* mice displayed slightly but significantly lower grip strength (Figure 3A; males 90.6%, females 92.5% of control F_(1,42)_ = 4.664; p = 0.038). With a subgroup of mice we performed a Rota-rod test at different speeds (16, 24, and 32 rpm; each for 5 min) and observed clear differences only at higher speed. When challenged for a longer period (10 min) on the Rota-rod at fixed speeds (24, 32 rpm), B6.NZO-*Nob3.38^N/N^* mice stayed longer on the rotating rod than B6.NZO-*Nob3.38^B/B^* mice. This effect is visible in male and female mice (Figure 3B) and becomes significant when we combine both groups (F_(1,42)_ = 5.729; p = 0.0212). Furthermore, in the exploration and anxiety examining mazes, NZO allele carriers displayed significantly different behaviors. B6.NZO-*Nob3.38^N/N^* mice spent less time (20%) in the illuminated compartment of the light-dark avoidance arena compared to the controls (31%, F_(1,42)_ = 7.574; p = 0.0087; Figure 3C) with less transitions between compartments (F_(1,42)_ = 6.425; p = 0.0151), remained longer in the open areas of the O-Maze, and stayed initially longer in the center of the open field (F_(1,42)_ = 5.258; p = 0.0269; Figure 3D). During fear conditioning, B6.NZO-*Nob3.38^N/N^* mice showed less freezing after the conditioned tone in a neutral environment (F_(1,41)_ = 5.426; p = 0.0248). The Morris Water Maze revealed that B6.NZO-*Nob3.38^B/B^* mice took more time to escape to the hidden platform (Figure 3E, left panel). However, the similar length of the path to the platform (data not shown) indicated that this was not due to a deficit in spatial learning but resulted from longer times of immobility and less swimming activity particularly of B6.NZO-*Nob3.38^B/B^* male mice (Figure 3E, right panel). Since several QTL on distal Chr. 1 associate with locomotor behavior, we analyzed locomotor-dependent parameter obtained in the individual tests. For the activity in the open field we observed a significantly (p = 0.016) elevated activity in female B6 allele carriers (*B/B*: 2356.6±193.6) in comparison to NZO allele carriers (*N/N*: 1681.2±174.6). Furthermore, evaluating the data of the first 5 min we observed that B6 carriers move faster and longer distances than NZO carriers, in the second interval both genotypes did not differ, whereas NZO carriers were more active in the third interval (10–15 min). These data indicate that the mice exhibit different exploration behavior. The activity in the O-Maze test was significantly different for B6 and NZO allele carriers when data of males and females were combined. Here we also see that B6 carriers are faster than NZO carriers (F_(1,39)_ = 4.304; p = 0.045). Presumably due to the low number of mice this difference is no longer significant when we calculate only for males or females. In the light-dark test we did not detect differences in respect to the distance and speed the mice moved in the light area. In summary, these data are consistent with the conclusion that *Nob3.38* not only modifies adiposity but also neurobehavioral traits influencing activity and anxiety.

**Figure 4:**
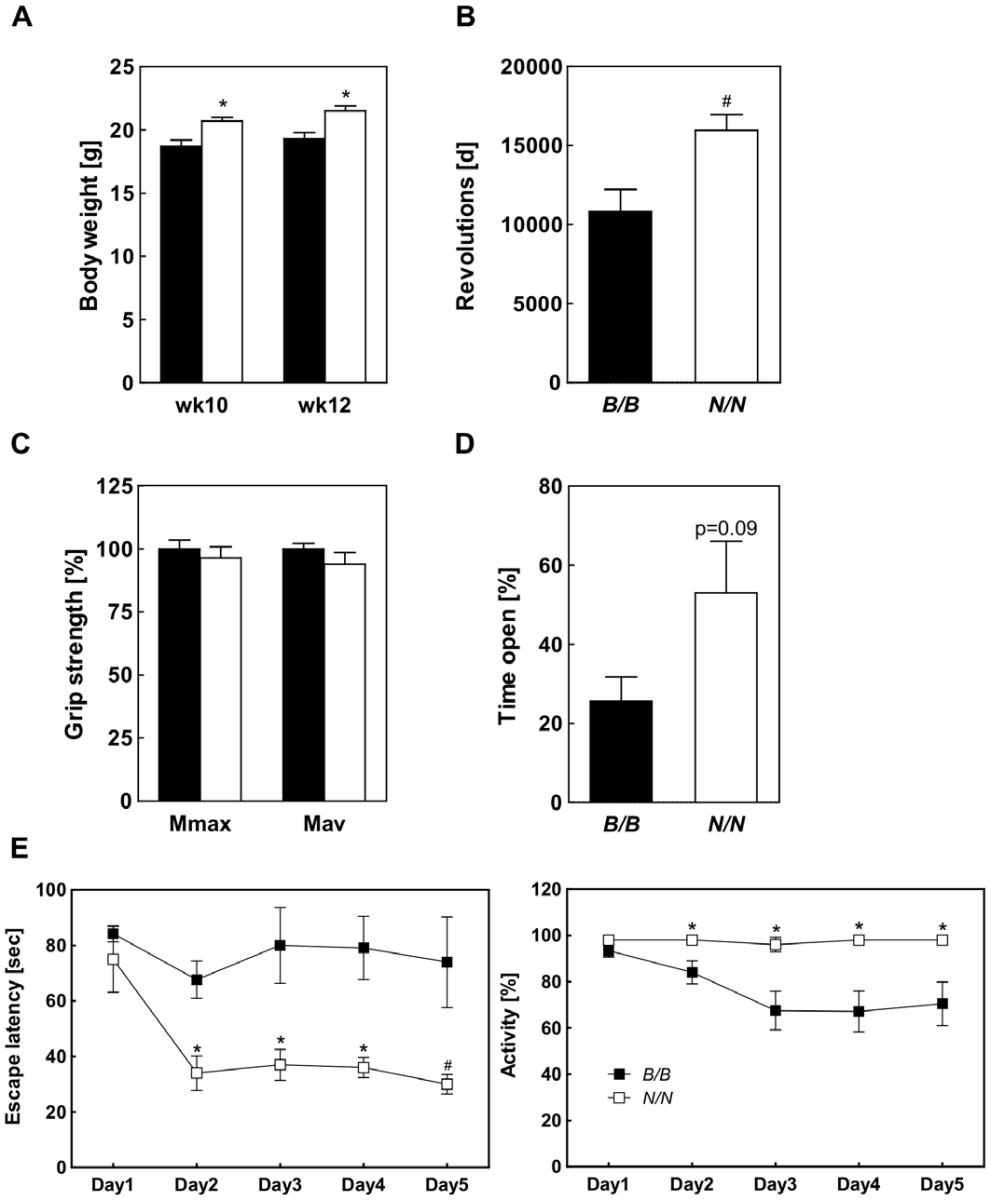
Characterization of the body weight and behavior of RCS-IX. (A) Body weight, (B) running wheel activity, (C) grip strength, and (D) preference on the O-Maze were compared between *B/B* and *N/N* allele carriers. (E) Spatial learning was assessed by the Morris Water Maze test and escape latency (left panel) and activity (right panel) were determined during acquisition (day 1–3) and reversal (day 4–5). For testing the subcongenic line RCS-IX 5 females of *B/B* and 7 females of *N/N* genotype were used. (#p<0.05; *p<0.01).

The *Nob3.38* consists of 38 Mbp which includes the critical interval of 2.2 Mbp that is responsible for elevated body weight of NZO allele carriers [24]. In order to test whether this interval also modifies behavior we tested a subcongenic line (RCS-IX) carrying 14.2 Mbp including *Qrr1* and the *Ifi200* locus (Figure 1) for running wheel activity, grip strength and Rota-rod performance, and in the open field, O-maze, water maze, and light-dark avoidance tests. We could confirm differences in body weight (Figure 4A). At both time points, at the age of 10 and of 12 weeks body weights of B6 allele carriers were significantly lower. Studying the running wheel activity at three consecutive days we observed an elevated voluntary activity for NZO carriers (Figure 4B). In contrast to B6.NZO-*Nob3.38^N/N^* mice above, the heavier NZO carriers did not show less grip strength (Figure 4C). The O-maze open area preference (Figure 4C) and the swimming behavior in the water maze (Figure 4D) validated significant differences between NZO and B6 allele carriers, indicating that these behavioral traits are dependent on the genes located in this shorter interval. Interestingly, for RCS-IX the difference was significant for females, whereas it was much smaller in male B6.NZO-*Nob3.38* mice (Figure 3E). In contrast, Rota-rod performance, behaviors in the open field and light-dark avoidance tests and freezing after fear conditioning were similar between the two groups (data not shown), indicating that further presumably more distal or proximal located gene(s) modify these phenotypes.

A haplotype map of the 14.2 Mb of RCS-IX displaying the polymorphic regions between strains (NZO/HILtJ, SJL/J, NZB/BINJ, DBA/2J, BALB/cJ, 129SvEvTac, C3H/HeJ, FVB/NJ) crossed with C57BL/6J, C57BL/10J, or CAST/EiJ in order to study behavioural phenotypes (listed in Tab. S2) is shown in Figure S1 and allows defining the critical interval carrying responsible genes. The map demonstrates that the relevant fragment has a size of 4.4 Mbp, between 173–177.4 Mbp including 112 genes. Within this part we have recently sequenced 31 genes of a 2.2 Mb interval (175.3–177.6) in NZO and B6 and discovered 96 SNPs resulting in amino acid exchanges in 23 genes. The most serious alteration in the sequenced regions was the microdeletion including a loss of function of one protein (Ifi202b) and presumably the disruption of other regulatory elements [24].

### Expression of Ifi202b in different mouse strains

**Figure 5:**
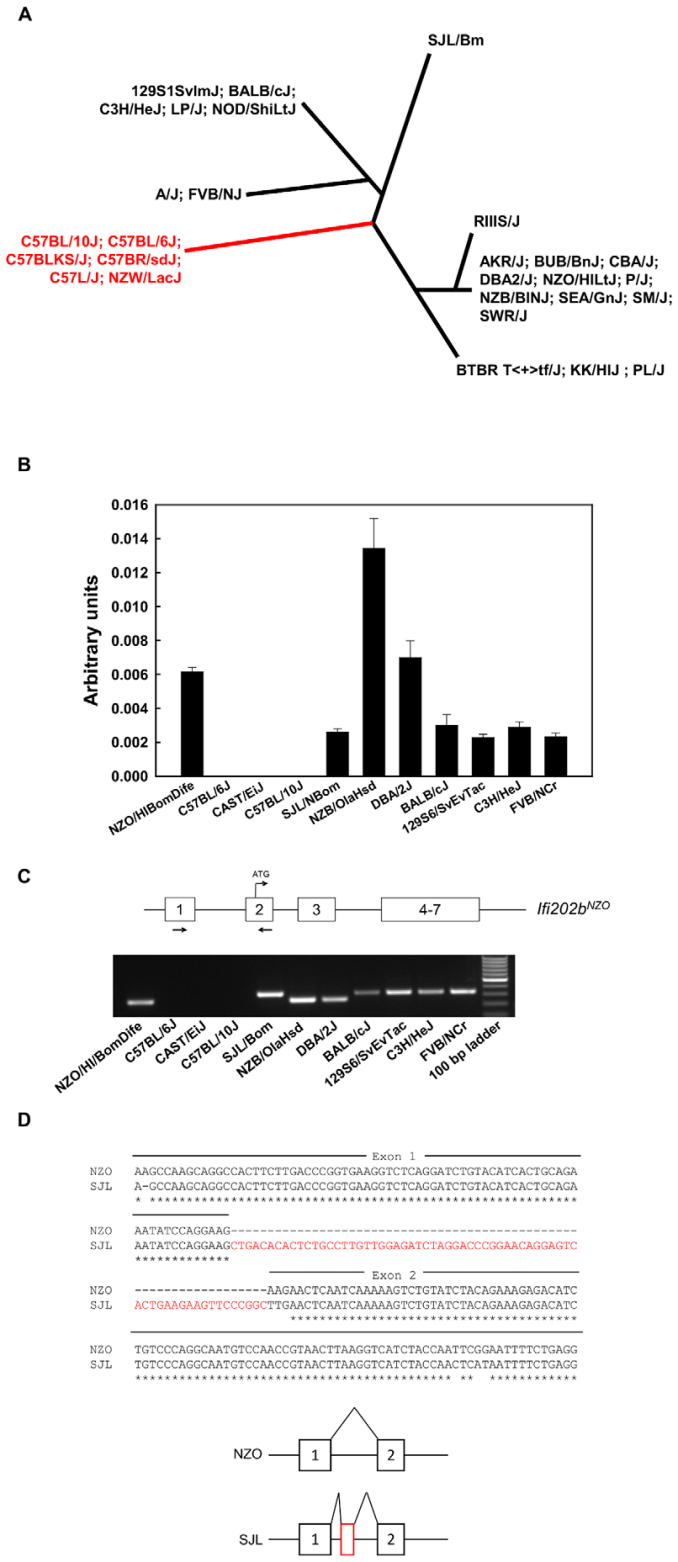
Expression of *Ifi202b* in different inbred strains of mice. (A) Strains in which *Ifi202b* is expressed as detected by microarray [36] are marked in black, lack of *Ifi202b* expression is marked in red. The figure shows a phylogenetic tree of 28 strains. It was generated by the Mouse Phylogeny Viewer (http://msub.csbio.unc.edu/) [37] based on SNP information of the segment 175,860,234–175,928,281 bp (Chr. 1, NCBI Build 37/mm9). Of these 28 strains, 22 express *Ifi202b*, whereas 6 do not. (B) Quantitative real time PCR performed on white adipose tissue of indicated mouse strains as described in the Method section. (C) PCR products received by amplification of *Ifi202b* cDNA (white adipose tissue) between exon 1 and exon 2 as indicated in the scheme. (D) The different size of the PCR product is the result of integration of 68 bp between the sequence of exon 1 and exon 2 as indicated in red. Sequence of SJL corresponds to EST clone M31418, sequence of NZO exhibits GenBank accession number BankIt1573081 JX945582.

The data described above indicate that the critical interval of *Nob3.38* between 173–177.4 Mbp including the microdeletion of the 5′-flanking region of the *Ifi202b* gene in B6 mice might be responsible for the diverse traits associated with *Qrr1*. In order to test whether the strains used for detection of linkage disequilibrium at Chr. 1 consistently differed in their expression of *Ifi202b* we used data by Gatti et al. [36] who had performed mRNA profiling in liver of inbred mice, allowing estimation which strains express *Ifi202b* and which do not. Figure 5 shows the mouse family tree of 28 strains which was generated by Mouse Phylogeny Viewer (http://msub.csbio.unc.edu/) [37] by a comparison of the segment 175,860,234–175,928,281 bp (Chr. 1, NCBI Build 37/mm9). Of these, 22 express *Ifi202b*, whereas 6 do not (Figure 5A). Several C57 strains and NZW/LacJ lack *Ifi202b* mRNA in liver, whereas close relatives of NZO (NZB/BINJ and KK/HIJ) express *Ifi202b*. We confirmed these results for some strains by performing qRT-PCR on gonadal adipose tissue and detected *Ifi202b* mRNA in NZB/OlaHsd, BALB/cJ, C3H/HeJ, 129S6/SvEvTac, FVB/NCrl, SJL/NBom, DBA/2J but not in C57BL/10J and CAST/EiJ (Figure 5B). Furthermore, we performed specific PCR with primers corresponding to exon 1 and exon 2 of *Ifi202b* on the cDNA from gonadal adipose tissue of several mouse strains and failed to detect a product in C57BL/6J, C57BL/10J, and CAS/EiJ mice (Figure 5C). In order to clarify the difference in the size of PCR products of SJL and NZO mice we sequenced the PCR product and observed an integration of 68 bp between the sequence of exon 1 and exon 2, indicating that in several mouse strains an additional exon is spliced between exon 1 and exon 2 (Figure 5D). The sequence of the SJL stains corresponds to the EST clone M31418.

### Screen for genes affected by the critical interval within Nob3.38

**Figure 6:**
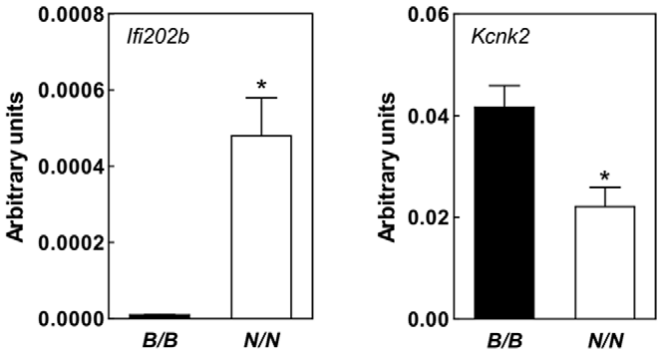
Expression of *Kcnk2* in cerebellum of B6.NZO-*Nob3.38^B/B^* and B6.NZO-*Nob3.38^N/N^* mice. Expression of *Kcnk2* was determined by qRT-PCR (B6.NZO-*Nob3.38^B/B^*: n = 7; B6.NZO-*Nob3.38^N/N^*: n = 8). (*p<0.005).

We recently performed transcriptome analysis in adipose tissue of B6.NZO-*Nob3.38^N/N^* and B6.NZO-*Nob3.38^B/B^* mice and found marked alteration of several mRNAs [24]. In order to identify potential candidates that might be responsible for the behavioral differences we screened array data that we received from peripheral tissues (liver, skeletal muscle; Table S3 and adipose tissue [24]) for transcripts that are described to be expressed in brain. As indicated in Table S3 a large number of genes are indeed also present in brain. We therefore screened those genes in respect to their impact on behavior. One important transcript which is highly expressed in the cerebellum [38] and influences depression when deleted [39] is the potassium channel *Knck2* (also designated TREK-1). *Knck2* exhibits a significantly lower expression in the cerebellum of B6.NZO-*Nob3.38^N/N^* than B6.NZO-*Nob3.38^B/B^* mice (Figure 6), which might indicate that this difference participates in a reduced excitability and the altered behavior. However, these results are based on transcriptome data from liver and muscle and it is likely that other or additional brain-specific genes of the interval are affected that were not detectable in peripheral tissues.

## Discussion

The present data demonstrate that recombinant congenic mice differing only in a small fragment of the obesity QTL *Nob3.38* on distal Chr. 1 also exhibit behavioral alterations in respect to activity, exploration, and anxiety. A C57BL/6J specific interval (173–177.4 Mbp) which includes a recently identified microdeletion overlaps with the QTL hotspot *Qrr1* [25] that associates with some behavioral phenotypes. The microdeletion includes a loss-of-function mutation of the transcriptional regulator *Ifi202b* which affects body weight by elevating the expression of *11β-HSD1* in adipose tissue [24]. Our data indicate that either the microdeletion or another variant within the critical interval influence several genes (e.g. *Dusp23*, *Pcp4l1*, *Fmn2*, *Rgs7*) in a cis-acting manner that may be responsible for the effects on neural functions and behavior.

Since the C57BL/6J strain was used in most of the intercrosses defining the *Qrr1* [25], we speculate that the deletion and/or other variants in the fragment of the B6 genome might be responsible not only for suppression of obesity but also for other traits associated with *Qrr1*. This assumption is supported by several points: (1) Our congenic strains which carry the microdeletion (B6.NZO-*Nob3.38^B/B^*) also exhibit neurobehavioral alterations in comparison to B6.NZO-*Nob3.38^N/N^* mice. Both lines differed in Rota-rod performance and in anxiety as previously demonstrated for instance in the 129S6/SvEvTac×C57BL/6J cross [34], in the DBA/2/J×C57BL/6 cross [40], or in the Balb/cJ×C57BL/6J cross [41] (2) Among nine parental mice that were crossed with C57BL/6 or C57BL/10 for QTL analysis that finally defined *Qrr1*, six were shown to contain the *Ifi202b* locus like NZO mice (Figure 5) [25]. (3) Similar to our data, strong differences in the *Ifi202b* expression were found in other C57BL/6J crosses. In an integrated analysis of a C57BL/6J×A/J F2 (B6AF2) cross Derry et al. [42] identified eQTL that showed strong effects for *Ifi202b* with LOD scores of 83.72 for liver and of 107.38 for adipose tissue [42]. In addition, in the C57BL/6×DBA/2J cross (BXD) an eQTL appears for *Ifi202b* (http://www.genenetwork.org). Moreover, recombinant BXD strains only show *Ifi202b* expression carrying the DBA allele: The expression value of the parental C57BL/6J is −0.116, for DBA 3.9 (http://www.genenetwork.org) and the interval-specific congenic line BXD6 lacks *Ifi202b* expression, whereas *Ifi202b* was detected in BXD8 (4.676), BXD29 (4.767), and BXD62 (4.601) which carry the DBA allele of the *Ifi200* cluster (http://www.genenetwork.org) [25].

Interestingly, not all characteristics that were obtained in the B6.NZO-*Nob3.38* line were also visible in the subcongenic line RCS-IX which carries 14.2 instead of 38 Mbp. The lower voluntary activity, the higher anxiety (O-maze), and reduced activity (water maze) of B6 allele carriers appear to be mediated by the microdeletion and/or variants in its close proximity that finally modulate expression of genes like *Dusp23* and *Pcp4l1* presumably in a cis-acting manner. It is also feasible that the microdeletion, which also affects the expression of *11β-Hsd1* [24] converting 11-dehydrocorticosterone to corticosterone, indirectly causes these pleiotropic behavioral effects by altering the glucocorticoid regulation [43]. Glucocorticoids are crucially involved in fundamental regulatory circuits and alterations have a variety of behavioral effects including depression, anxiety, and stress. In contrast, some behavioral aspects e. g. the reduced endurance of *Nob3.38^B/B^* mice as detected by Rota-rod performance is not induced by the lack of *Ifi202b*, it appears to be mediated by a gene(s) upstream or downstream of the 14.2 Mbp fragment.

There are several possibilities to explain the behavior differences between B6 and NZO allele carriers: The phenotype is the consequence (1) of the lack of *Ifi202b*, (2) of a peripheral signal which is released in response to Ifi202b action, (3) of the microdeletion which does not only disrupt parts of the *Ifi202b* gene but also other regulatory elements, or (4) of a combination of variants within the critical fragment that contribute to the behavioral traits produced by the locus. Ifi200 proteins are described to associate with inhibition of cell proliferation, modulation of apoptosis, and cell differentiation [44]. Thus, it might be possible that the effects of Ifi202b on cell proliferation and differentiation could also be responsible for the complex behavioral traits of *Qrr1*. However, since the expression of *Ifi202b* in the brain, e.g. in the cortex, cerebellum, hippocampus, bulbus olfactorius is much lower than in peripheral tissues like adipose tissue or skeletal muscle (about 1%; data not shown) it is unlikely that *Ifi202b* mediates the observed behavioral alterations directly. Some data indicate that the microdeletion or the critical interval of *Nob3.38* exhibit cis-acting capacity to influence expression of other genes that are expressed in brain and that are therefore better candidates to be responsible for different behaviour. Expression profiling of peripheral tissues (liver and skeletal muscle) – which does not necessarily include all neuronal genes relevant for the phenotype - from B6.NZO-*Nob3.38^B/B^* and B6.NZO-*Nob3.38^N/N^* mice identified alterations of genes located in proximity to the deletion (e.g. *Dusp23*, *Pcp4l1*, *Rgs5*) which were not affected by overexpression of *Ifi202b* in skeletal muscle (data not shown). In particular, the genes *Fmn2*, *Rgs7*, *Dusp23*, and *Pcp4l1* were recently discussed as strong candidates in *Qrr1* [25]; we found that these genes are highly expressed in the brain. Two of them are differentially expressed in B6.NZO-*Nob3.38^B/B^* and B6.NZO-*Nob3.38^N/N^* mice. For example, *Rgs7* exhibits a two to three fold higher expression in NZO allele carrier than in B6 allele carriers (data not shown). Another example is *Kcnk2* which exhibits a significantly decreased expression in the cerebellum of NZO allele carriers in comparison to B6 allele carriers (Figure 6). This effect might at least participate in the lower anxiety of B6.NZO-*Nob3.38^N/N^* mice. *Kcnk2* encodes for a two-pore-domain potassium channel and belongs to a class of channels that participate in the mediation of excitability of individual neurons. They are open at membrane potentials in physiological conditions and believed to contribute to the background or leak currents setting the resting potential [45], [46]. Kcnk2 is regulated by 5-hydroxytryptamine (5-HT) that is involved in learning and memory [47] and responds to stressful stimuli [47], [48]. The fact that *Kcnk2^−/−^* mice are much less susceptible to induced ‘depression-like’ states [39] might indicate that B6.NZO-*Nob3.38^N/N^* mice show less fear due to its lower *Kcnk2* expression in the cerebellum. However, *Kcnk2* is located on Chr.1 at 191 Mbp, within *Nob3.38*, but outside of the critical interval of RCS-IX (165.4–179.6 Mbp). In fact studying the *Kcnk2* expression in cerebellum of RCS-IX we did not detect differences between B6 and NZO allele carriers (relative expression of 1.01±0.17 in B6 versus 1.16±0.06 in NZO). Furthermore, testing *Kcnk2* expression after overexpression of *Ifi202b* did not show differences (relative expression of mock: 0.224±0.04 vs. *Ifi202b* infected: 0.229±0.09). This indicates that *Kcnk2* mediates phenotypes like anxiety (light/dark avoidance) and coordination (Rota-rod) that did show up in RCS-IX. As a consequence of this, the variants within the region 173–177.4 Mp including the microdeletion and/or cis-regulatory effects are responsible for increased running wheel activity and exploration behavior in the open field, but not for anxiety and coordination.

The discovery of a microdeletion as genetic cause of complex traits like obesity and behavior is not surprising. One prominent example is the human Prader-Willi syndrome which presents both metabolic and neuronal alterations. A 5–7 Mb deletion of the paternally inherited chromosomal 15q11.2–q13 region is responsible for (1) a neurobehavioral disorder manifested by infantile hypotonia and (2) feeding difficulties in infancy, followed by morbid obesity secondary to hyperphagia [49]. Recently, a 593 kb, rare (0.7%) deletion on Chr. 16p11.2 was shown to be significantly (p = 6.4×10^−8^) enriched in obese patients compared to controls [50], [51], demonstrating the potential importance of rare variants with strong effects on body weight. The frequency of 16q11.2 deletions was higher (2.9%) in cohorts including developmental and cognitive disabilities [52], supporting the strong correlation between neuronal phenotypes and obesity [53], [54]. Another 2.3 Mb deletion was recently identified on Chr. 11p14.1 as an important cause of neurobehavioral abnormalities and obesity. This deletion encompasses the *BDNF* and *LIN7C* genes that are implicated in the regulation of development and differentiation of neurons and synaptic transmission [55]. However, our studies have shown that the *Nob3.38* interval between 173–177.4 Mbp of NZO mice that induces obesity is in the opposite direction with the obese human subjects because it mediates higher activity and lowered anxiety traits.

In summary, our data present a C57BL/6J specific interval, which carries a microdeletion and several SNPs that protect from adiposity and modulate behavioral phenotypes.

## Supporting Information

Figure S1 Haplotype map displaying the polymorphic regions of strains (NZO/HILtJ, SJL/J, NZB/BINJ, DBA/2J, BALB/cJ, 129S6/SvEvTac, C3H/HeJ, FVB/NJ; depicted in color) crossed with C57BL/6J, C57BL/10J, or CAST/EiJ for studying behavioral phenotypes. The map is based on MDA data set (MPD∶CGD-MDA1) information. The SNP data were from Mouse Diversity Genotyping Array, 550,000 locations for 123 strains of mice. Mouse Phenome Database web site, The Jackson Laboratory, Bar Harbor, Maine USA. http://phenome.jax.org, Sept, 2012.
(TIF)

Table S1 Primer and probe sequences (NCBI Build 37/mm9).
(DOC)

Table S2 QTL linked to behavioral traits on Chr. 1 from 172–178 Mbp (adapted from Mouse Genome Informatics and Mozhui et al., 2008).
(DOC)

Table S3 Gene expression profiling in liver (A) and skeletal muscle (B) derived from the congenic line *Nob3.38*. Genes described to be expressed in brain are marked with B.
(DOCX)
