# Supplementary figures and images for: An Interval of the Obesity QTL Nob3.38 within a QTL Hotspot on Chromosome 1 Modulates Behavioral Phenotypes
Source: PLoS One. 2013 Jan 4;8(1):e53025. doi: 10.1371/journal.pone.0053025 (PMC3537729; doi:10.1371/journal.pone.0053025)

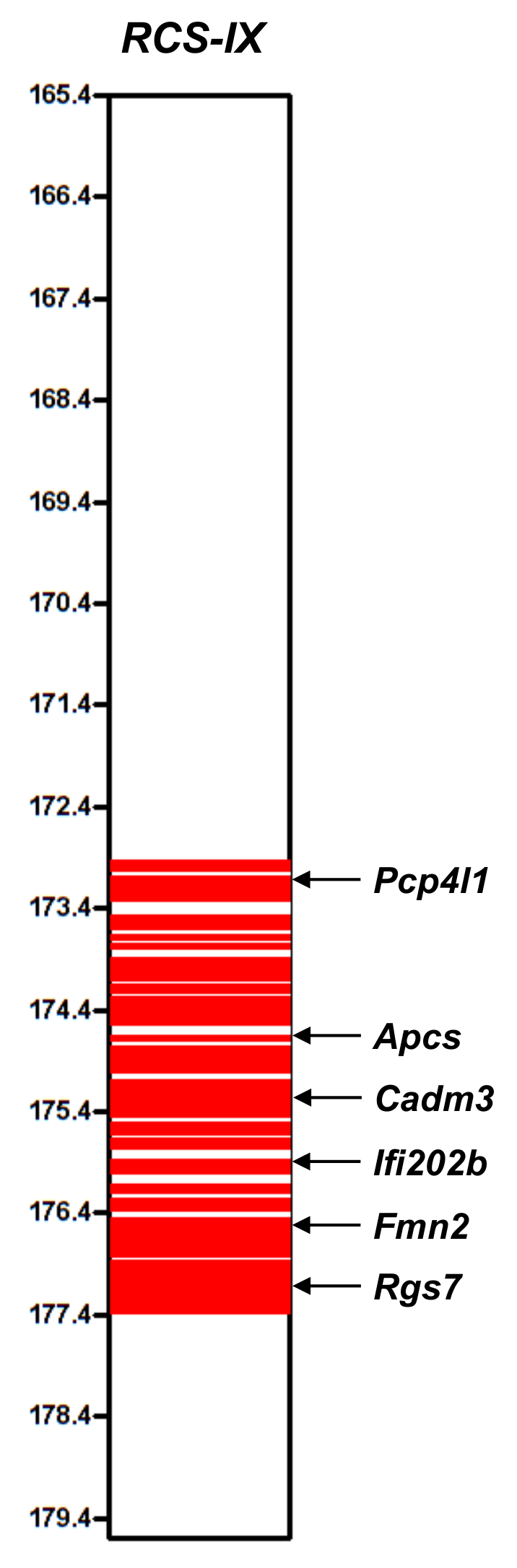

Supplement: Figure S1 — Haplotype map displaying the polymorphic regions of strains (NZO/HILtJ, SJL/J, NZB/BINJ, DBA/2J, BALB/cJ, 129S6/SvEvTac, C3H/HeJ, FVB/NJ; depicted in color) crossed with C57BL/6J, C57BL/10J, or CAST/EiJ for studying behavioral phenotypes. The map is based on MDA data set (MPD∶CGD-MDA1) information. The SNP data were from Mouse Diversity Genotyping Array, 550,000 locations for 123 strains of mice. Mouse Phenome Database web site, The Jackson Laboratory, Bar Harbor, Maine USA. http://phenome.jax.org, Sept, 2012. (TIF) [file pone.0053025.s001.tif]
