# Supplementary material for: An Interval of the Obesity QTL Nob3.38 within a QTL Hotspot on Chromosome 1 Modulates Behavioral Phenotypes
Source: PLoS One. 2013 Jan 4;8(1):e53025. doi: 10.1371/journal.pone.0053025 (PMC3537729; doi:10.1371/journal.pone.0053025)
Supplement: Table S1 — Primer and probe sequences (NCBI Build 37/mm9). (DOC) [file pone.0053025.s002.doc]

Table S1: Primer and probe sequences (NCBI Build 37/mm9)

Microsatellite markers for genotyping of the different congenic lines

| **Congenic line** | **Primer** | **Position [bp]** | **5´-*Primer* (Fwd)** | **3´-*Primer* (Rev)** |
| --- | --- | --- | --- | --- |
| ***Nob3.38*** | **D1Mit202** | 158971080 | CCA TAA GCC TCC TCT  TTC CC | AAA ATG AAC TCA GCG  GGT TG |
|  | **D1Mit209** | 193317066 | TCC ATC CAT ACT CCT  GTC TGC | CAA GGA CTA GGG CTG  TCA CTG |
| **RCS-IX** | **D1Mit143** | 165430952 | CTC AAG GAT ATA CTG  GAT TCA TGT G | TAT GGT GCT TCA AAG  ATA TAG ATA TGG |
|  | **D1Mit115** | 179610205 | AAG GGA ATG GAA TTA  GGG TCA | TAA CGG ACA CCC ATT  TTA AAC A |

Primer and dual labeled probes for expression analysis

| **Gene** | **Accession number** | **5´-*Primer* (Fwd)** | **3´-*Primer* (Rev)** |
| --- | --- | --- | --- |
| *Ifi202b* | NM_008327 | CAA CTA TGA GAA AGG  AGA TAA ACT CCA A | GTT TCA TCA AGG GAG AAA AGC TAC TAA |
|  |  | Probe: TTC ACC TGA GAA AAG GAA ATG GGA AAC CA | |
| *Actb* | NM_007393 | GCC AAC CGT GAA AAG ATG AC | TAC GAC CAG AGG CAT ACA G |
|  |  | Probe: TTG AGA CCT TCA ACA CCC CAG CCA | |

Primer for the analysis of the genomic organization of *Ifi202b*

| **5´-*Primer* (Fwd)** | **3´-*Primer* (Rev)** |
| --- | --- |
| CCC TCT TCC TTT ACA CCC AAC | GCC TGG GAC AGA TGT CTC TT |
