# Supplementary material for: An Interval of the Obesity QTL Nob3.38 within a QTL Hotspot on Chromosome 1 Modulates Behavioral Phenotypes
Source: PLoS One. 2013 Jan 4;8(1):e53025. doi: 10.1371/journal.pone.0053025 (PMC3537729; doi:10.1371/journal.pone.0053025)
Supplement: Table S2 — QTL linked to behavioral traits on Chr. 1 from 172–178 Mbp (adapted from Mouse Genome Informatics and Mozhui et al., 2008). (DOC) [file pone.0053025.s003.doc]

Table S2:

QTL linked to behavioral traits on Chr. 1 from 172-178 Mbp (adapted from Mouse Genome Informatics and Mozhui et al., 2008).

| **Symbol** | **Trait** | **Cross** | **Reference** |
| --- | --- | --- | --- |
| *Actre1* | Activity response to ethanol 1 | BALB/cJxC57BL/6J | Demarest et al. [56] |
|  |  | C57BL/6JxDBA/2J | Hitzemann et al.[57] |
|  |  | C57BL/6JxLP/J | Malmanger et al [58] |
| *Alcdp1* | Alcohol dependency 1 | C57BL/6JxDBA/2J | Buck et al. [59] |
| *Alcw1* | Alcohol withdrawal 1 | C57BL/6JxDBA/2J | Crabbe, 1996 [60] |
| *Bslm4* | Basal locomotor activity 4 | BALB/cJxC57BL/6J |  |
|  |  | C57BL/6JxDBA/2J | Hitzemann et al., [57] |
|  |  | C57BL/6JxLP/J |  |
| *Eila1* | Ethanol induced locomotor activity 1 | C3H/HeJxC57BL/6J | Downing et al. [61] |
| *Emo1* | Emotionality 1 | BALB/cJxC57BL/6J | Turri et al. [41] |
| *Hpic2* | Haloperidol induced catalepsy 2 | C57BL/6JxDBA/2J | Patel et al. [62] |
| *Pbw1* | Pentobarbital withdrawal QTL 1 | C57BL/6JxDBA/2J | Buck et al. [59] |
| *Pbwm* | Pentobarbital withdrawal modifier | C57BL/6JxDBA/2J | Hood et al. [63] |
| *Rrodp1* | Rotarod performance 1 | 129S6/SvEvTacxC57BL/6J | Kelly et al. [34] |
| *Elnt* | Escape latencies during navigation task | C57BL/6JxDBA/2J | Milhaud et al., [40] |
