# Supplementary material for: An Interval of the Obesity QTL Nob3.38 within a QTL Hotspot on Chromosome 1 Modulates Behavioral Phenotypes
Source: PLoS One. 2013 Jan 4;8(1):e53025. doi: 10.1371/journal.pone.0053025 (PMC3537729; doi:10.1371/journal.pone.0053025)
Supplement: Table S3 — Gene expression profiling in liver (A) and skeletal muscle (B) derived from the congenic line Nob3.38. Genes described to be expressed in brain are marked with B. (DOCX) [file pone.0053025.s004.docx]

**Table S3**: Gene expression profiling in liver (A) and skeletal muscle (B) derived from the congenic line *Nob3.38*. Genes described to be expressed in brain are marked with B.

**A**

| **Gene Symbol** |  | **Target Id** | **log2FoldChange** | **p-Value** |
| --- | --- | --- | --- | --- |
| *Ifi202b* |  | NM_011940 | 9.58 | 2.7E-07 |
| *EG545391* |  | AK029617 | 4.14 | 4.7E-07 |
| *LOC545383* |  | ENSMUST00000097462 | 2.15 | 1.9E-05 |
| *Darc* | B | NM_010045 | 2.04 | 1.2E-04 |
| *Crp* |  | NM_007768 | 1.87 | 4.2E-07 |
| *Hhipl1* |  | AK166269 | 1.74 | 8.0E-03 |
| *Fmn2* | B | NM_019445 | 1.56 | 3.7E-03 |
| *Apcs* |  | NM_011318 | 1.48 | 1.2E-04 |
| *AK047917* |  | AK047917 | 1.39 | 1.8E-02 |
| *2410076I21Rik* |  | AK010725 | 1.37 | 4.4E-02 |
| *AK038048* |  | AK038048 | 1.29 | 1.1E-02 |
| *Dlgap1* |  | NM_177639 | 1.28 | 3.1E-02 |
| *Lrrc16a* |  | AK051570 | 1.27 | 4.8E-02 |
| *Dppa3* |  | NM_139218 | 1.26 | 2.5E-02 |
| *Lrit2* |  | NM_173418 | 1.24 | 2.3E-02 |
| *1810023F06Rik* |  | NM_145449 | 1.22 | 2.0E-02 |
| *Olfr1260* |  | NM_146981 | 1.21 | 5.5E-03 |
| *AK043928* |  | AK043928 | 1.19 | 2.3E-03 |
| *AK037068* |  | AK037068 | 1.18 | 2.9E-02 |
| *Abcc10* |  | NM_145140 | 1.14 | 9.8E-03 |
| *Adamts6* |  | AK030315 | 1.13 | 7.6E-03 |
| *Exoc4* | B | AK038125 | 1.12 | 1.0E-02 |
| *Kcnj9* | B | NM_008429 | 1.10 | 7.0E-03 |
| *Dusp8* | B | NM_008748 | 1.10 | 7.8E-03 |
| *NAP041487-1* |  | NAP041487-1 | 1.09 | 2.7E-02 |
| *AK054519* |  | AK054519 | 1.08 | 9.9E-03 |
| *6530404N21Rik* |  | BC052355 | 1.08 | 4.1E-02 |
| *Ifi203* |  | AK080088 | 1.08 | 1.2E-02 |
| *F11r* |  | NM_172647 | 1.07 | 1.0E-05 |
| *AI132487* |  | NM_001012310 | 1.06 | 2.9E-02 |
| *2610028H24Rik* |  | NM_029816 | 1.04 | 4.4E-02 |
| *AK044348* |  | AK044348 | 1.04 | 5.1E-03 |
| *AK048646* |  | AK048646 | 1.03 | 2.1E-03 |
| *Gm131* |  | NM_001111143 | 1.02 | 4.5E-02 |
| *Olfr494* |  | NM_146737 | 1.02 | 2.1E-02 |
| *Rnf38* |  | NM_175201 | 1.01 | 1.2E-02 |
| *AK080322* |  | AK080322 | 1.00 | 7.6E-03 |
| *Rexo4* |  | AK079733 | 1.00 | 3.6E-02 |
| *Cyp2s1* |  | NM_028775 | -1.01 | 8.4E-03 |
| *Myo15b* |  | ENSMUST00000093911 | -1.01 | 1.1E-02 |
| *Dnm3* | B | NM_001038619 | -1.03 | 3.2E-02 |
| *Zfp831* |  | NM_001099328 | -1.03 | 1.0E-02 |
| *Pcp4l1* | B | NM_025557 | -1.03 | 2.1E-02 |
| *M19902* |  | M19902 | -1.04 | 4.1E-02 |
| *Nnat* | B | NM_010923 | -1.05 | 2.2E-02 |
| *Flrt3* | B | NM_178382 | -1.07 | 3.6E-02 |
| *Tubb2b* | B | NM_023716 | -1.09 | 3.8E-02 |
| *Degs2* |  | NM_027299 | -1.11 | 2.0E-02 |
| *Spock2* | B | NM_052994 | -1.11 | 5.0E-02 |
| *NAP058430-1* |  | NAP058430-1 | -1.11 | 1.6E-02 |
| *AB041806* |  | AB041806 | -1.15 | 1.6E-02 |
| *Rasgrf1* | B | BC042449 | -1.15 | 4.5E-02 |
| *A730017C20Rik* | B | AK048493 | -1.16 | 2.5E-02 |
| *Srp9* | B | AK003606 | -1.16 | 5.2E-04 |
| *Abp1* |  | NM_029638 | -1.17 | 2.8E-03 |
| *Prp2* | B | M19419 | -1.20 | 2.3E-02 |
| *Muc4* |  | NM_080457 | -1.20 | 4.6E-02 |
| *Raver2* | B | NM_183024 | -1.21 | 1.8E-02 |
| *Batf3* |  | NM_030060 | -1.21 | 1.2E-03 |
| *Ints7* |  | AK020029 | -1.21 | 1.6E-03 |
| *S100b* | B | NM_009115 | -1.22 | 2.6E-03 |
| *Samd12* | B | NM_177225 | -1.24 | 1.3E-03 |
| *Krt19* |  | NM_008471 | -1.26 | 2.7E-03 |
| *Slc9a2* |  | AK077026 | -1.31 | 3.1E-02 |
| *Tmem45b* |  | NM_144936 | -1.31 | 6.5E-03 |
| *Klf5* |  | NM_009769 | -1.32 | 3.6E-03 |
| *1110032A04Rik* |  | NM_133675 | -1.32 | 4.4E-02 |
| *Tspan1* |  | NM_133681 | -1.33 | 2.7E-03 |
| *A530016L24Rik* |  | NM_177039 | -1.39 | 3.5E-02 |
| *9430083B18Rik* |  | AK020503 | -1.40 | 2.7E-02 |
| *Angptl1* |  | NM_028333 | -1.40 | 2.0E-02 |
| *Tmem54* |  | NM_025452 | -1.42 | 1.3E-02 |
| *Ddr2* |  | NM_022563 | -1.42 | 2.5E-05 |
| *Slamf8* |  | NM_029084 | -1.43 | 1.5E-04 |
| *TC1614884* |  | TC1614884 | -1.45 | 7.7E-03 |
| *9030617O03Rik* |  | AK081256 | -1.53 | 2.8E-02 |
| *Lrrc26* |  | NM_146117 | -1.54 | 5.2E-03 |
| *Mmp7* |  | NM_010810 | -1.55 | 1.5E-02 |
| *Ckmt1* | B | NM_009897 | -1.59 | 1.9E-03 |
| *Fam83e* |  | NM_001033170 | -1.62 | 3.2E-02 |
| *Rd3* |  | NM_023727 | -1.75 | 2.7E-03 |
| *CA481501* |  | CA481501 | -1.77 | 1.7E-02 |
| *Tm4sf5* |  | NM_029360 | -1.79 | 1.9E-02 |
| *Fcgbp* |  | NM_001122603 | -1.79 | 1.1E-02 |
| *Tmc5* |  | NM_028930 | -1.90 | 4.8E-02 |
| *Lgals2* |  | NM_025622 | -2.00 | 1.4E-03 |
| *Cyp2c65* |  | AK008688 | -2.16 | 2.5E-02 |
| *Duoxa2* |  | NM_025777 | -2.19 | 2.7E-03 |
| *Cyp2c66* |  | NM_001011707 | -2.30 | 4.8E-02 |
| *3110045C21Rik* |  | AK014177 | -2.41 | 4.9E-03 |
| *Spink4* |  | NM_011463 | -2.52 | 3.3E-03 |
| *1700009P17Rik* |  | NM_001081275 | -2.71 | 2.9E-07 |
| *EG240921* |  | XM_136331 | -3.05 | 1.0E-05 |
| *Ifi205* |  | NM_172648 | -3.05 | 1.2E-03 |
| *Tlr5* |  | AF186107 | -3.22 | 1.4E-06 |
| *Ifi203* |  | NM_001045481 | -3.34 | 8.1E-08 |
| *Mnda* |  | NM_001033450 | -3.53 | 1.1E-05 |
| *Susd4* | B | NM_144796 | -3.93 | 9.0E-04 |
| *Lefty1* |  | NM_010094 | -4.46 | 1.6E-04 |
| *BB200491* |  | BB200491 | -5.27 | 4.6E-06 |

**(p≤0.05 and log2 fold change ≥1.0 or ≤-1.0)**

**B**

| **Gene Symbol** |  | **Target Id** | **log2FoldChange** | **p-Value** |
| --- | --- | --- | --- | --- |
| *Ifi202b* |  | NM_011940 | 10.18 | 3.1E-03 |
| *Hmga2-ps1* |  | AK033703 | 3.72 | 6.5E-04 |
| *AK145002* |  | AK145002 | 2.93 | 4.2E-03 |
| *A330023F24Rik* |  | AK030377 | 2.27 | 2.0E-03 |
| *AK010841* |  | AK010841 | 1.78 | 2.0E-06 |
| *4930430O22Rik* |  | AK019590 | 1.65 | 3.6E-05 |
| *Ptpn5* | B | NM_013643 | 1.64 | 4.9E-02 |
| *Kcnk2* | B | NM_010607 | 1.61 | 2.5E-02 |
| *ENSMUST00000114683* |  | ENSMUST00000114683 | 1.58 | 8.6E-05 |
| *Ear7* |  | NM_017385 | 1.56 | 4.9E-02 |
| *Slfn8* |  | NM_181545 | 1.55 | 7.1E-03 |
| *Olfr741* |  | NM_207133 | 1.51 | 4.8E-02 |
| *EG545391* |  | AK029617 | 1.50 | 1.4E-02 |
| *Bcl11a* | B | AK122557 | 1.46 | 2.5E-02 |
| *Magee1* |  | NM_053201 | 1.44 | 5.6E-04 |
| *Tcstv3* |  | NM_153523 | 1.39 | 2.1E-02 |
| *Ifi203* |  | AK172505 | 1.39 | 1.1E-05 |
| *NAP071351-1* |  | NAP071351-1 | 1.39 | 4.8E-02 |
| *Cd80* |  | BC131959 | 1.32 | 1.5E-02 |
| *Ccdc36* |  | NM_001135198 | 1.29 | 2.6E-02 |
| *EG665317* |  | XM_979163 | 1.27 | 4.5E-03 |
| *Gpr20* | B | NM_173365 | 1.26 | 3.0E-02 |
| *AK034754* |  | AK034754 | 1.26 | 4.4E-02 |
| *Cspg4* | B | AK052394 | 1.26 | 2.2E-06 |
| *Krt20* |  | NM_023256 | 1.26 | 2.8E-02 |
| *100040392* |  | XM_001474438 | 1.25 | 1.4E-02 |
| *Akap13* |  | AK006382 | 1.21 | 1.1E-02 |
| *Galnt9* | B | NM_198306 | 1.21 | 2.7E-02 |
| *Slfn9* |  | NM_172796 | 1.19 | 2.8E-02 |
| *EG236749* |  | NM_001034864 | 1.19 | 4.4E-03 |
| *Gcnt1* |  | NM_010265 | 1.19 | 4.8E-02 |
| *Ifi203* |  | AK080088 | 1.17 | 8.8E-04 |
| *AK085324* |  | AK085324 | 1.16 | 1.8E-02 |
| *BC048671* |  | NM_177738 | 1.15 | 4.3E-02 |
| *Zp3* |  | NM_011776 | 1.15 | 4.4E-02 |
| *Hsd17b3* |  | NM_008291 | 1.14 | 4.4E-02 |
| *Adra2b* | B | NM_009633 | 1.11 | 2.1E-02 |
| *NAP026497-1* |  | NAP026497-1 | 1.09 | 1.1E-02 |
| *Epsti1* |  | NM_178825 | 1.09 | 3.2E-02 |
| *Gpr62* | B | BY714829 | 1.08 | 2.3E-02 |
| *A_52_P1037106* |  | A_52_P1037106 | 1.07 | 8.2E-03 |
| *Zfp251* |  | NM_001007568 | 1.06 | 1.5E-02 |
| *Chit1* | B | NM_027979 | 1.06 | 1.9E-02 |
| *AK033258* |  | AK033258 | 1.04 | 1.6E-03 |
| *AK031195* |  | AK031195 | 1.01 | 6.8E-04 |
| *ENSMUST00000072475* |  | ENSMUST00000072475 | 1.01 | 4.2E-02 |
| *Casq1* |  | NM_009813 | 1.00 | 2.0E-04 |
| *Dpt* |  | NM_019759 | 1.00 | 5.9E-03 |
| *Epha1* | B | NM_023580 | 1.00 | 3.4E-02 |
| *Ttr* | B | NM_013697 | -1.00 | 4.3E-02 |
| *Tat* |  | NM_146214 | -1.02 | 3.2E-02 |
| *Gcgr* | B | L38613 | -1.03 | 7.6E-03 |
| *NAP018073-001* |  | NAP018073-001 | -1.04 | 1.5E-02 |
| *Lgals7* |  | NM_008496 | -1.06 | 8.9E-03 |
| *Gulo* |  | NM_178747 | -1.07 | 4.4E-02 |
| *Cyp2d10* |  | NM_010005 | -1.08 | 3.3E-02 |
| *Tm4sf4* |  | NM_145539 | -1.10 | 2.1E-02 |
| *Dnase1l3* |  | NM_007870 | -1.10 | 4.4E-02 |
| *Pot1a* |  | AK005318 | -1.11 | 5.3E-03 |
| *Sult1d1* |  | NM_016771 | -1.11 | 4.3E-02 |
| *1700009P17Rik* |  | NM_001081275 | -1.12 | 5.5E-06 |
| *Sh3rf2* | B | NM_172966 | -1.13 | 7.0E-03 |
| *Krt28* |  | AK014642 | -1.13 | 2.8E-02 |
| *Actrt2* |  | NM_028513 | -1.15 | 1.7E-02 |
| *Lrrc52* | B | NM_001013382 | -1.16 | 3.7E-02 |
| *Cyp2c70* |  | NM_145499 | -1.17 | 4.9E-02 |
| *AK038954* |  | AK038954 | -1.19 | 2.1E-03 |
| *Ints7* |  | AK020029 | -1.20 | 4.9E-04 |
| *Hrg* |  | NM_053176 | -1.21 | 3.8E-02 |
| *Cyp3a25* |  | NM_019792 | -1.22 | 3.7E-03 |
| *Hal* |  | NM_010401 | -1.22 | 4.1E-02 |
| *Rp1h* |  | AK044612 | -1.22 | 4.9E-02 |
| *Cyp2b9* |  | NM_010000 | -1.22 | 2.1E-02 |
| *Fetub* |  | NM_021564 | -1.23 | 2.2E-02 |
| *Apob* |  | NM_009693 | -1.23 | 1.8E-02 |
| *AK036293* |  | AK036293 | -1.23 | 4.2E-02 |
| *1300017J02Rik* |  | NM_027918 | -1.24 | 3.4E-02 |
| *Grem2* |  | NM_011825 | -1.25 | 6.2E-04 |
| *Klk1b16* |  | NM_008454 | -1.27 | 5.0E-02 |
| *Saa4* | B | NM_011316 | -1.27 | 3.2E-02 |
| *Clec4f* |  | NM_016751 | -1.28 | 4.8E-02 |
| *NAP112858-1* |  | NAP112858-1 | -1.29 | 1.0E-03 |
| *Abcg5* |  | NM_031884 | -1.31 | 1.2E-02 |
| *TC1711584* |  | TC1711584 | -1.31 | 3.9E-02 |
| *E130202H07Rik* |  | AK053684 | -1.33 | 2.2E-02 |
| *Ugt2b37* |  | NM_053215 | -1.33 | 2.1E-02 |
| *Ppox* |  | NM_008911 | -1.33 | 1.4E-06 |
| *Ccnb1ip1* |  | NM_001111119 | -1.35 | 1.1E-02 |
| *Serpinc1* |  | NM_080844 | -1.38 | 3.3E-02 |
| *Pipox* |  | NM_008952 | -1.40 | 2.2E-02 |
| *Mnda* |  | NM_001033450 | -1.41 | 1.4E-03 |
| *Slc27a5* |  | NM_009512 | -1.41 | 2.6E-02 |
| *Apoh* | B | NM_013475 | -1.44 | 3.2E-02 |
| *NAP058430-1* |  | NAP058430-1 | -1.45 | 8.2E-05 |
| *AK016486* |  | AK016486 | -1.49 | 4.1E-04 |
| *Apon* |  | NM_133996 | -1.51 | 4.3E-02 |
| *Rasal2* | B | AK086196 | -1.54 | 1.2E-04 |
| *1810008I18Rik* |  | AK050412 | -1.55 | 8.4E-03 |
| *Synpr* | B | NM_028052 | -1.57 | 4.3E-02 |
| *Hsd17b2* |  | NM_008290 | -1.59 | 3.8E-02 |
| *Srp9* | B | AK003606 | -1.60 | 4.4E-04 |
| *Cyp3a44* |  | NM_177380 | -1.62 | 1.5E-02 |
| *Cyp2d26* |  | NM_029562 | -1.63 | 3.1E-02 |
| *Tmprss6* |  | NM_027902 | -1.63 | 1.5E-02 |
| *Gltpd2* |  | NM_146020 | -1.63 | 2.9E-02 |
| *Pcp4l1* | B | NM_025557 | -1.64 | 2.6E-04 |
| *Apom* | B | NM_018816 | -1.64 | 1.0E-02 |
| *Serpinf2* |  | NM_008878 | -1.64 | 1.5E-02 |
| *Rgn* |  | NM_009060 | -1.65 | 3.8E-02 |
| *Proc* |  | NM_001042768 | -1.66 | 9.2E-03 |
| *1190003J15Rik* |  | AK004470 | -1.66 | 9.8E-03 |
| *Akr1c6* |  | NM_030611 | -1.67 | 4.4E-02 |
| *Mst1* | B | NM_008243 | -1.69 | 2.6E-02 |
| *Cyp4f15* |  | NM_134127 | -1.70 | 4.0E-02 |
| *Gc* |  | NM_008096 | -1.72 | 4.6E-02 |
| *Cpb2* |  | ENSMUST00000022576 | -1.72 | 3.4E-02 |
| *Rdh7* |  | NM_017473 | -1.72 | 3.5E-02 |
| *Slco1b2* |  | NM_178235 | -1.75 | 4.0E-02 |
| *Ifi205* |  | NM_172648 | -1.75 | 3.0E-04 |
| *Plg* |  | NM_008877 | -1.76 | 3.6E-02 |
| *Ftcd* |  | NM_080845 | -1.76 | 3.8E-02 |
| *Itih3* |  | NM_008407 | -1.77 | 1.4E-02 |
| *382044* |  | NM_001081372 | -1.78 | 1.5E-02 |
| *Serpina3k* |  | NM_011458 | -1.83 | 4.3E-02 |
| *Akr1c20* |  | BC021607 | -1.85 | 4.0E-02 |
| *G6pc* | B | NM_008061 | -1.87 | 3.2E-02 |
| *St8sia3* | B | NM_009182 | -1.89 | 2.6E-02 |
| *Uroc1* |  | NM_144940 | -1.91 | 3.0E-02 |
| *Cyp3a41a* |  | NM_017396 | -1.91 | 2.3E-02 |
| *2010003K15Rik* |  | XM_900828 | -1.98 | 1.7E-02 |
| *Serpina1d* |  | NM_009246 | -2.00 | 3.9E-02 |
| *C4bp* |  | NM_007576 | -2.00 | 2.0E-02 |
| *Dpys* |  | ENSMUST00000110306 | -2.01 | 9.6E-03 |
| *Cyp2j5* |  | NM_010007 | -2.01 | 2.4E-02 |
| *Mug2* |  | NM_008646 | -2.01 | 1.5E-02 |
| *Cyp4f14* | B | NM_022434 | -2.01 | 3.7E-03 |
| *Serpina1c* |  | NM_009245 | -2.01 | 4.0E-02 |
| *Kng1* | B | NM_023125 | -2.03 | 3.4E-02 |
| *Cyp2c29* | B | NM_007815 | -2.03 | 4.0E-02 |
| *Abcb11* | B | NM_021022 | -2.04 | 2.0E-02 |
| *Gckr* | B | NM_144909 | -2.07 | 4.5E-03 |
| *EG13909* |  | NM_144511 | -2.12 | 2.8E-02 |
| *Apoa1* |  | NM_009692 | -2.13 | 4.9E-02 |
| *Bhmt2* | B | NM_022884 | -2.15 | 2.3E-02 |
| *Ahsg* |  | NM_013465 | -2.15 | 1.7E-02 |
| *NAP001160-001* |  | NAP001160-001 | -2.17 | 3.5E-02 |
| *Mug1* |  | NM_008645 | -2.18 | 1.7E-02 |
| *Serpina6* | B | NM_007618 | -2.20 | 2.9E-02 |
| *Hpd* |  | NM_008277 | -2.22 | 2.7E-02 |
| *Proz* | B | AK005011 | -2.25 | 1.5E-02 |
| *Hamp2* | B | NM_183257 | -2.25 | 6.8E-03 |
| *1190003J15Rik* |  | NM_029821 | -2.30 | 1.1E-02 |
| *Cfi* | B | NM_007686 | -2.31 | 4.1E-02 |
| *F2* |  | NM_010168 | -2.32 | 1.4E-02 |
| *Serpina1b* |  | BC037008 | -2.33 | 3.8E-02 |
| *Fgg* |  | NM_133862 | -2.34 | 9.0E-03 |
| *Dmgdh* | B | NM_028772 | -2.36 | 4.9E-02 |
| *Fgb* |  | NM_181849 | -2.36 | 1.2E-02 |
| *F12* |  | NM_021489 | -2.39 | 2.7E-02 |
| *BC089597* |  | NM_145424 | -2.39 | 1.4E-02 |
| *Hc* |  | NM_010406 | -2.39 | 4.1E-04 |
| *Mat1a* |  | NM_133653 | -2.39 | 4.0E-02 |
| *Aadac* |  | NM_023383 | -2.40 | 4.1E-02 |
| *Tlr5* | B | AF186107 | -2.43 | 2.0E-05 |
| *Pzp* |  | NM_007376 | -2.46 | 4.9E-02 |
| *Fbp1* | B | NM_019395 | -2.47 | 4.8E-02 |
| *Es1* |  | NM_007954 | -2.47 | 2.1E-02 |
| *Apof* |  | NM_133997 | -2.49 | 2.0E-02 |
| *Fga* |  | NM_010196 | -2.49 | 4.4E-02 |
| *Mbl2* | B | NM_010776 | -2.50 | 1.8E-02 |
| *Ambp* | B | NM_007443 | -2.52 | 4.4E-02 |
| *Cps1* |  | AK028683 | -2.58 | 1.6E-02 |
| *Agxt* |  | NM_016702 | -2.64 | 2.2E-02 |
| *Igfbp1* |  | NM_008341 | -2.80 | 1.7E-02 |
| *Afm* | B | NM_145146 | -2.82 | 1.8E-02 |
| *Lefty1* |  | NM_010094 | -3.22 | 6.8E-06 |
| *3110045C21Rik* |  | AK014177 | -3.82 | 1.5E-06 |
| *BB200491* |  | BB200491 | -5.51 | 1.2E-07 |

**(p≤0.05 and log2 fold change ≥1.0 or ≤-1.0)**
